# Two cases of fatal methemoglobinemia caused by self-poisoning with sodium nitrite: A case report

**DOI:** 10.1097/MD.0000000000028810

**Published:** 2022-02-18

**Authors:** Sung Hoon Mun, Gwan Jin Park, Ji Han Lee, Young Min Kim, Hyun Seok Chai, Sang Chul Kim

**Affiliations:** Department of Emergency Medicine, Chungbuk National University Hospital, Cheongju, Korea 776, 1st Sunwhan-ro, Seowon-gu, Cheongju-si, Chungcheongbuk-do, Korea.

**Keywords:** methemoglobinemia, methylene blue, sodium nitrite, suicide

## Abstract

**Rationale:**

Sodium nitrite intoxication reportedly causes severe methemoglobinemia. Recent studies reported that most clinically significant cases resulted from intentional exposure in suicidal attempts. We describe 2 cases of severe methemoglobinemia secondary to intentional sodium nitrite intoxication in suicidal attempts.

**Patients concerns:**

A 26-year-old man and 20-year-old woman attempted suicide by taking sodium nitrite, and were brought to the emergency department.

**Diagnosis:**

The male patient collapsed at the scene. He ingested approximately 18 g of sodium nitrate, and his methemoglobin level was 90.3%. The female patient was conscious, but was cyanotic. She ingested approximately 12.5 g of sodium nitrite, and her methemoglobin level was 54.6%.

**Interventions:**

The male patient received advanced cardiac life support in the emergency department. Methylene blue was immediately administered for the female patient.

**Outcomes:**

The male patient died despite aggressive resuscitation. The female patient's cyanosis resolved, and her methemoglobin level decreased to 1.2% 3 hours later.

**Lessons:**

The immediate administration of methylene blue in severe methemoglobinemia patients prevented fatal consequences. The public should be informed about the accessibility and toxicity of sodium nitrite.

## 1 Introduction

Sodium nitrite is a coloring agent or food preservative. It is also used medically to treat cyanide poisoning and adjunct to disinfect fluids.^[1]^ Sodium nitrite intoxication can cause severe methemoglobinemia. Methemoglobinemia is a rare but potentially lethal condition wherein hemoglobin oxidized to methemoglobin and becomes unable to bind and transport oxygen. Most cases of clinically significant sodium nitrite intoxication were accidental, such as inadvertent intake.^[^2^,^^3^^]^ However, recent studies have reported intentional intake in suicide attempts.^[^4^,^^5^^]^

Regardless of the etiology, the severity of methemoglobinemia depends on the methemoglobin concentration. A methemoglobin concentration above 10% causes cyanosis. Methemoglobin levels greater than 50% induce comas, seizures, and death.^[6]^ Methylene blue is used to treat symptomatic patients with methemoglobin levels greater than 20% or patients with risk factors, such as anemia or congestive heart failure. The symptoms are expected to improve immediately after methylene blue administration.^[1]^ This report describes the 2 cases of severe methemoglobinemia due to sodium nitrite poisoning in a companion suicidal attempt.

## 2 Case presentation

### 2.1 Case 1: fatality case

A 26-year-old man, who had a cardiac arrest after taking sodium nitrate in a suicidal attempt, was brought to the emergency department (ED) by the Emergency Medical Services (EMS) ambulance. He had ingested approximately 18 g of sodium nitrite dissolved in a beverage. He was soon unconscious, and a suicide accomplice called the EMS. The initial rhythm was asystole at the scene. Standard advanced cardiac life support was provided, but the patients died in the ED. His methemoglobin concentration was 90.3%.

### 2.2 Case 2: survival case

**Figure 1:**
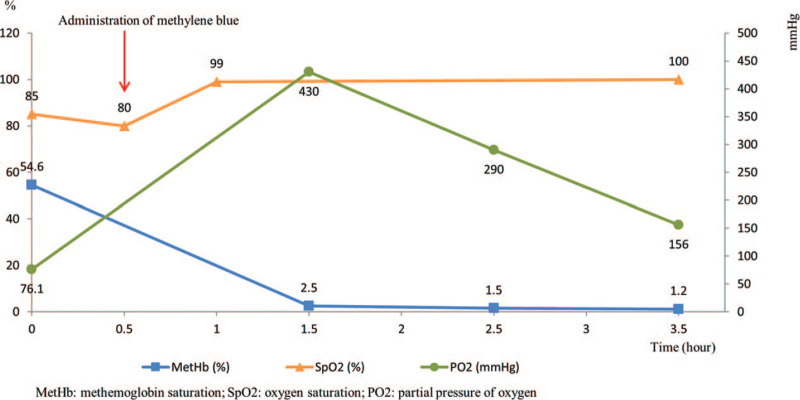
Serial changes in the percentage of methemoglobin, oxygen saturation, partial pressure of oxygen obtained from pulse oximetry and arterial blood gas analysis.

A 20-year-old woman presented to the ED after intentionally ingesting sodium nitrite to attempt suicide. She ingested approximately 12.5 g of sodium nitrite dissolved in a beverage. When the EMS arrived at the scene, she vomited repeatedly, and had an initial reading on pulse oximeter was 86%. In the ED, she was alert but was dyspneic and cyanotic. Her initial methemoglobin level was 54.6%. Methylene blue (1 mg/kg) was administered 30 minutes after her arrival at the ED. Her symptoms rapidly improved within an hour after methylene blue administration, and the methylene blue levels decreased to 1.2% 3 hours later (Fig. 1). A psychiatric interview was conducted 8 hours after she arrived at the ED, and the patient was discharged from the ED that afternoon.

### 2.3 Ethics statement

Written informed consent was obtained from a legally authorized representative(s) for anonymized patient information to be published in this article. This study was approved by the Institutional Review Board of Chungbuk National University Hospital (IRB No. 2021-03-031).

## 3 Discussion

Sodium nitrite is a commercially available coloring agent, food preservative, and medical agent. Sodium nitrite intoxication due to accidental intake or suicidal attempts causes severe methemoglobinemia. Nitrite induces toxicity by oxidizing ferrous (Fe^2+^) to ferric (Fe^3+^) hemoglobin, producing methemoglobin. Methemoglobin is unable to carry oxygen and reduces oxygen delivery to tissues. It also aggravates cellular hypoxia by altering the oxygen-hemoglobin dissociation curve.^[3]^

Cyanosis is a consistent physical finding in patients with substantial methemoglobinemia. At a methemoglobin level of 20% to 50%, patients experience dizziness, syncope, dyspnea, exercise intolerance, fatigue, headache, and weakness. Methemoglobin levels above 50% cause dysrhythmia, central nervous system depression, coma, metabolic acidosis, seizure, and tachypnea.^[3]^ Methemoglobin levels >70% are lethal, but survival has been reported in more than 90% of cases.^[^5^,^^7^^]^

Methylene blue is the first-line antidote for severe methemoglobinemia, and the standard dose is 1 to 2 mg/kg intravenously over 5 minutes.^[1]^ The methemoglobin level decreases significantly within 1 to 2 hours after a single dose. In this case, the methemoglobin concentration significantly decreased after a single administration of methylene blue as Figure 1 suggested. Our research institute was designated as the regional hospital to provide various antidotes like a poison center in the US. Thus, it was immediately administered to our patient, who had a good prognosis.

However, other studies have reported the difficult diagnosis and treatment of methemoglobinemia, particularly when the causative substance is unknown, or when it is mistaken for other critical illnesses. Methemoglobinemia should be considered in patients presenting with cyanosis or desaturation of unknown cause.^[^8^,^^9^^]^ Health care providers should be aware of the toxicity of sodium nitrite, especially in large amounts.

The number of cases of sodium nitrite intoxication due to a suicidal attempt has increased.^[^4^,^^5^^]^ A recent study conducted in Korea reported 14 autopsy cases from sodium nitrite poisoning from 2013 to 2019. Until 2017, there has been 1 autopsy every year, but it increased to 8 cases in 2018. Teenagers and adults in their thirties were the most common (57.1%) victims, and 78.6% of total cases were confirmed or suspected suicide cases.^[10]^

Individuals using sodium nitrite to attempt suicide easily obtain and share information s regarding these suicide methods online.^[4]^ This is a significant public health issue since sodium nitrite is fatal when taken excessively. The public should be informed about its potential dangers, and online information dissemination regarding suicide methods should be limited. In the United Kingdom, individuals who want to handle the sodium nitrite must have the appropriate licenses by law.^[11]^

## 4 Conclusion

We reported 2 cases of severe methemoglobinemia secondary to intentional sodium nitrite intake in suicidal attempts. Health care providers should be aware of the toxicity of sodium nitrite. Early administration of methylene blue prevented fatal outcomes. Public efforts are needed since sodium nitrite is readily available, but it is lethal when taken excessively in suicide attempts.

## Author contributions

**Conceptualization:** Sung Hoon Mun, Gwan Jin Park.

**Data curation:** Sung Hoon Mun, Gwan Jin Park.

**Formal analysis:** Sung Hoon Mun, Gwan Jin Park.

**Investigation:** Sung Hoon Mun.

**Methodology:** Gwan Jin Park.

**Writing – original draft:** Sung Hoon Mun, Gwan Jin Park.

**Writing – review & editing:** Gwan Jin Park, Ji Han Lee, Young Min Kim, Hyun Seok Chai, Sang Chul Kim.
